# Effects of UV Irradiation and Storage on the Performance of Inverted Red Quantum-Dot Light-Emitting Diodes

**DOI:** 10.3390/nano11061606

**Published:** 2021-06-18

**Authors:** Yu Luo, Junjie Wang, Pu Wang, Chaohuang Mai, Jian Wang, Boon Kar Yap, Junbiao Peng

**Affiliations:** 1 State Key Laboratory of Luminescent Materials and Devices, Institute of Polymer Optoelectronic Materials and Devices, South China University of Technology, Guangzhou 510640, China; luoyulucas@163.com (Y.L.); wangjunjie20200928@163.com (J.W.); 201710102477@mail.scut.edu.cn (C.M.); jianwang@scut.edu.cn (J.W.); 2 School of Materials Science and Engineering, Guilin University of Electronic Technology, Guilin 541000, China; 18145717332@163.com; 3 The International School of Advanced Materials, School of Material Science and Engineering, South China University of Technology, Guangzhou 510640, China; boonkar@scut.edu.cn; 4 Electronic and Communications Department, Institute of Sustainable Energy, College of Engineering, University Tenaga Nasional, Kajang 43000, Malaysia

**Keywords:** quantum-dot, inverted QLEDs, UV irradiation, storage

## Abstract

We report the effects of ultraviolet (UV) irradiation and storage on the performance of ZnO-based inverted quantum-dot light-emitting diodes (QLEDs). The effects of UV irradiation on the electrical properties of ZnO nanoparticles (NPs) were investigated. We demonstrate that the charge balance was enhanced by improving the electron injection. The maximum external quantum efficiency (EQE) and power efficiency (PE) of QLEDs were increased by 26% and 143% after UV irradiation for 15 min. In addition, we investigated the storage stabilities of the inverted QLEDs. During the storage period, the electron current from ZnO gradually decreased, causing a reduction in the device current. However, the QLEDs demonstrated improvements in maximum EQE by 20.7% after two days of storage. Our analysis indicates that the suppression of exciton quenching at the interface of ZnO and quantum dots (QDs) during the storage period could result in the enhancement of EQE. This study provides a comprehension of the generally neglected factors, which could be conducive to the realization of high-efficiency and highly storage-stable practical applications.

## 1. Introduction

Quantum-dot light-emitting diodes (QLEDs) are promising large-area electroluminescent devices used for display and solid-state lighting applications, due to their high efficiency, tunable color, high color purity, and simple yet cost-effective solution processibility [1,2,3]. In the past few years, the performance of QLEDs has been significantly improved via thorough study of the core/shell structure of QDs [4,5], the surface ligands of QDs [6,7], and device structure engineering [8,9]. The QLEDs with high efficiency and long operational lifetime mainly adopt a multilayer hybrid structure with an organic hole injection/transport layer and an inorganic electron injection/transport layer (ZnO or ZnMgO). At present, the peak external quantum efficiency (EQE) of red, green, and blue QLEDs reach 30.9% [10], 23.9% [11], and 19.8% [12], respectively. Meanwhile, the extrapolated *T*_50_ operational lifetime (time taken for the luminance to drop to 50% of the initial luminance of 100 cd/m^2^) of red and green QLEDs has exceeded 1 million hours [5,9,10,13].

Many researchers have reported on conventional QLEDs with high operation stability but low storage stability. Recently, a great deal of attention has been paid to a fascinating phenomenon—namely, positive ageing behavior in conventional QLEDs [14,15,16]. This positive ageing behavior exhibits improvements in device efficiency and electrical conductance within several days of storage. Currently, the underlying origin of the positive ageing effect in QLEDs is still under debate. However, all the current research results agree that the positive ageing effect is related to the defect passivation of the inorganic electron transport layer ZnO, which can be caused by the reaction of ZnO with the acid in UV-curable resin [16] or with the evaporated electrode Al [15]. Although the performance of the device is improved after storage for a few days, the storage stability declines. This poor storage stability is unacceptable for practical applications.

We believe that an inverted device structure will effectively reduce the positive ageing effect caused by UV-curable acidic resin or electrode Al because the ZnO deposited on the bottom will be well-covered by the QDs layer and the organic layer. This will help to improve the storage stability. In this study, the UV curing light in the encapsulation process is shown to affect the performance and storage stability of inverted QLEDs. The UV irradiation desorbs the oxygen adsorbed on the surface of ZnO NPs and excites electrons from defect states, thereby significantly increasing the conductivity of ZnO and reducing the electron injection barrier to promote the balance of carrier injection. With an appropriate UV irradiation time of 15 min, the peak EQE and PE of the QLEDs were increased by 26% and 143%, respectively, compared with the values of the pristine device. Subsequently, we explored the performance changes of the inverted encapsulated device during the storage period. We found that the current density of the device gradually declined during the storage process, possibly resulting from the declining effect of UV light on the electron injection. However, the ZnO was still self-passivated at the bottom layer, which may be related to the adsorbed water on the surface of ZnO NPs and/or the QDs. The trace amount of water passivated the defect state of ZnO during storage and reduced the quenching of QDs, thus improving the efficiency of QLEDs.

## 2. Materials and Methods

### 2.1. Materials

The ITO glass substrates were purchased from China Southern Glass Holding Corp. (Shenzhen, China). The ZnO NPs solution was purchased from Guangdong Poly Photoelectric Technology Corp. (Jiangmen, China). The red nanocrystals (CdSe/ZnS with a typical solution PLQY of ~82%) were purchased from Suzhou Mesolight Inc. (Suzhou, China). The QDs solution had a pure red emission at 632 nm with a full width at half maximum (FWHM) of 26 nm, and the electroluminescence (EL) peak of QLEDs was located at 634 nm with an FWHM of 27 nm. The 4,4′,4″-tris(carbazol-9-yl)-triphenylamine (TcTa) and 4,4′-bis(N-carbazolyl)-1,1′-biphenyl (CBP) were purchased from Xi’an Polymer Light Technology Corp. (Xi’an, China). The molybdenum oxide (MoO_x_, 99.97%) was purchased from Sigma-Aldrich (St. Louis, MO, USA).

### 2.2. Device Fabrication

The glass/ITO substrates were thoroughly and sequentially cleaned in an ultrasonic bath with acetone, isopropanol, detergent, deionized water, and isopropanol, before being dried in a vacuum baking oven at 70 °C. After cleaning, the ITO substrates were transferred to a N_2_-filled glovebox to fabricate each functional layer. Firstly, the ZnO NPs were dissolved in ethanol at a concentration of 30 mg/mL, spin-coated on the ITO substrate at 3000 rpm for 30 s, and annealed at 150 °C for 15 min. Subsequently, the QDs which were dissolved in octane at a concentration of 20 mg/mL were spin-coated on the ZnO layer at 3000 rpm for 45 s and annealed at 120 °C for 12 min. After that, the samples were transferred to a vacuum evaporator (base pressure 9 × 10^−5^ Pa) for deposition of the organic layer and Al electrode. The deposition rates of TcTa, CBP, and MoO_x_ layers were 0.1–1 Å/s, and the deposition rate of Al electrode was 0.3–1.5 Å/s. The evaporation rates were monitored by a frequency counter. Then, devices were encapsulated using a glass cover plate with ultraviolet-curable epoxy and irradiated with a UV curing machine (EC-500, Electro-Lite Corporation, USA). The UV curing machine had four 365 nm lamps with a power of 9 W. The encapsulated devices, after UV irradiation for 5 min, were stored in a nitrogen-filled glovebox (with a gas concentration of O_2_ < 2 ppm and H_2_O < 0.1 ppm).

### 2.3. Characterization

The film thickness was determined by a step profiler (Bruker Dektak XT, Karlsruhe, Germany). The current density–voltage–luminance (J–V–L) characteristics were measured via a Keithley 236 source meter (Cleveland, OH, USA) and a silicon photodiode system, and calibrated by a Konica Minolta Chroma Meter CS-200 (Tokyo, Japan). The electroluminescence (EL) spectra, photoluminescence (PL) spectra, and photoluminescence quantum yield (PLQY) were measured by a QE Pro spectrometer system (Biaoqi Optoelectronics, Guangzhou, China). Kelvin probe measurements were carried out using the KP Technology SKP5050 system (KP Technology Ltd., Wick, UK) in a nitrogen-filled glovebox. Absorption spectra were obtained using the UV-2600 (SHIMADZU, Kyoto, Japan). Time-resolved photoluminescence (TRPL) spectra were measured using the Hamamatsu C11367-11 (Hamamatsu Photonics, Iwata, Japan). The EQE was calculated from the data of luminance, current density, and EL spectrum.

## 3. Results and Discussion

### 3.1. The Effects of UV Irradiation on Inverted QLEDs

**Figure 1:**
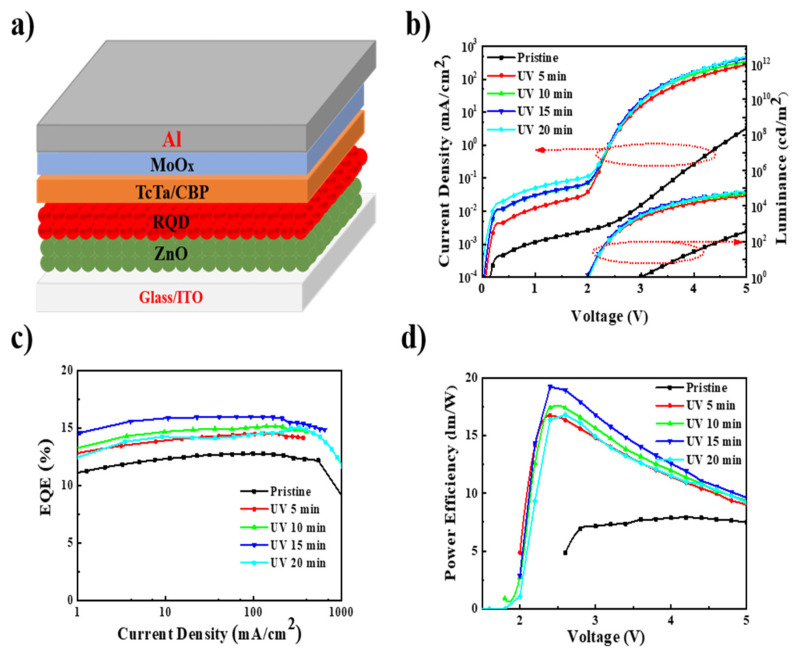
(**a**) Schematic structure of inverted QLEDs; (**b**) the current density–voltage–luminance (J–V–L) characteristics; (**c**) external quantum efficiency–current density (EQE–J) characteristics; (**d**) and power efficiency–voltage (PE–V) characteristics of the QLEDs treated with UV irradiation for different durations.

Inverted QLEDs with the hybrid structure of ITO/ZnO (60 nm)/QDs (25 nm)/TcTa (50 nm)/CBP (5 nm)/MoO_x_ (8 nm)/Al were fabricated. Figure 1a exhibits the schematic device structure of QLEDs. The ZnO, TcTa/CBP and MoO_x_ were used as the electron-transport layer, hole-transport layer, and hole-injection layer, respectively. The fresh devices, without UV-curable resin, were put into the UV curing machine and subjected to varying UV irradiation times. The UV light treatment and assessment of device characteristics were carried out in a nitrogen-filled glovebox.

With different UV irradiation times, the corresponding devices exhibited distinct current density–voltage–luminance (J–V–L), external quantum efficiency–current density (EQE–J), and power efficiency–voltage (PE–V) characteristics, as shown in Figure 1b–d. The characteristics of the devices are summarized in Table 1. The typical pristine device exhibited a low current density and high turn-on voltage (V_on_, 3.0 V), whereas the devices exposed to UV irradiation all had higher current density and lower V_on_ (2.0 V). For example, the pristine device showed a current density of 3.2 mA/cm^2^ at 5 V. However, the devices subjected to 15 min of UV irradiation had a current density of 426.9 mA/cm^2^ at 5 V, which is 133-fold that of the pristine device. The V_on_ of devices with UV irradiation were 1 V lower than that of the pristine device. These results indicate that UV irradiation improves the carrier injection of QLEDs. Moreover, after being subjected to 15 min of UV irradiation, all devices exhibited higher EQE and PE. The performance of the devices subjected to 15 min of UV irradiation demonstrated the most significant improvement, whereby EQE was enhanced by 26%, from 12.7% to 16.0%, and PE was greatly enhanced by 143%, from 7.9 lm/W to 19.2 lm/W. However, the leakage current of QLEDs was also continuously enhanced with increasing UV irradiation time, which may have originated from the promotion of electron accumulation at the interface of QDs and TcTa. With further increases in the UV irradiation time, the efficiency of the devices decreased, possibly due to unbalanced charge injections caused by the further increase in electron current.

**Figure 2:**
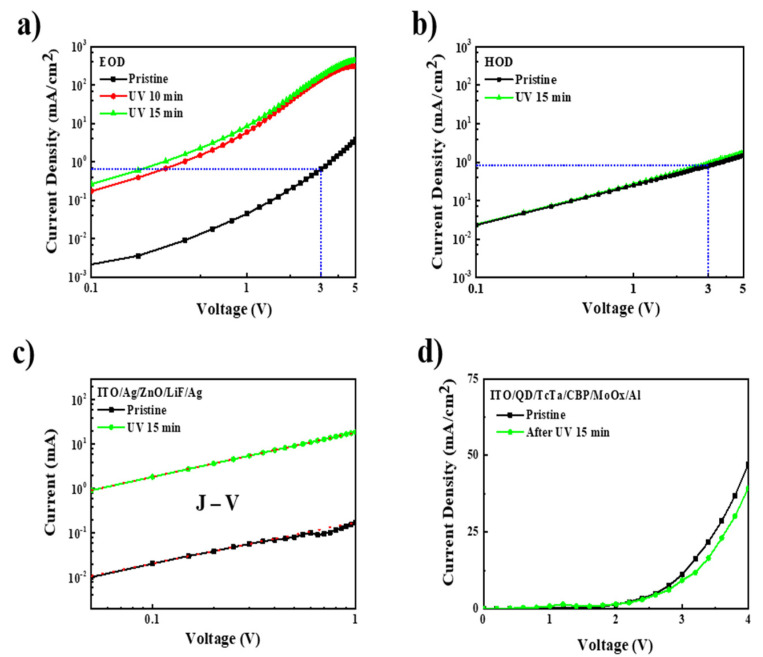
(**a**) J–V curves for electron-only devices (ITO/ZnO/QDs/ZnO/Al); (**b**) J–V curves for hole-only devices (ITO/PEDOT/TFB/QDs/TcTa/CBP/MoO_x_/Al); (**c**) J–V characteristics of electron devices (ITO/Ag/ZnO/LiF/Ag). The red dotted line exhibits the ohmic region of J–V; (**d**) J–V curves for the device without a ZnO layer.

To explain the origin of the effects of UV irradiation, we first investigated the J–V of electron-only devices (EODs) and hole-only devices (HODs) before and after UV irradiation. As shown in Figure 2a, the electron current density of EOD evidently improved after UV irradiation. For example, the pristine device showed a current density of 3.8 mA/cm^2^ at 5 V but the device after 15 min of UV irradiation exhibited a higher current density of 435.3 mA/cm^2^ at 5 V, which is almost a 113-fold enhancement compared to the value of the pristine device. In contrast, the current density of HOD was almost unchanged after UV irradiation. These results indicate that the enhancement of current in QLEDs is mainly due to the improvement of electron injection. In addition, we found that the hole current of the pristine HOD was higher than the electron current of the pristine EOD from 0–3 V, which illustrates that the hole current was more dominant in the pristine QLEDs. The very low electron current results in a high turn-on voltage, low current density, and low EQE because of the unbalanced charge in the pristine QLEDs. Moreover, in order to analyze the electron current enhancement of ZnO after UV irradiation, we tested the conductivity of ZnO film. The J–V characteristics of the ZnO thin-film are shown in Figure 2c. At the ohmic contact region (J–V), the conductivity of the ZnO film after UV irradiation was 4.3 × 10^−4^ S/mm, which was enhanced by 80-fold compared to the conductivity of pristine film, which was 5.4 × 10^−6^ S/mm. Therefore, the current enhancement of EOD is mainly ascribed to the increase in bulk conductivity of ZnO after UV irradiation. To further confirm that the current enhancement was solely related to the UV irradiation effect on ZnO, the J–V characteristics of the device without the ZnO layer (ITO/QD/TcTa/CBP/MoO_x_/Al) were measured. As shown in Figure 2d, the device without the ZnO layer had no current elevation after UV irradiation, which confirms that the current enhancement in QLEDs solely originated from the improved conductivity of ZnO after UV irradiation.

**Figure 3:**
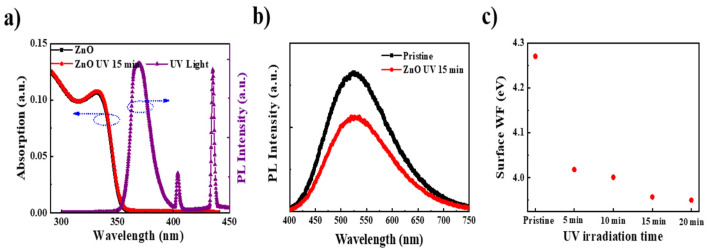
(**a**) Absorption of the ZnO film before and after 15 min of UV irradiation, and the emission spectrum of the UV curve machine; (**b**) PL spectra of the ZnO film before and after 15 min of UV irradiation in the UV–visible range excited by a 280 nm laser; (**c**) surface WF of the ZnO film on the ITO substrate with different UV irradiation times chartered by the SKP5050 system.

The effect of UV irradiation on the conductivity of ZnO is frequently attributed to adsorbed oxygen. Trace amounts of O_2_ could be adsorbed on the surface of ZnO NPs with a high surface-to-volume ratio despite the ZnO NPs being spun and annealed inside the glovebox, where O_2_ concentration might be just several ppm. The physically adsorbed O_2_ might turn into chemically adsorbed ions (such as O^−^ and O^2−^) by capturing the free electrons of ZnO, thus reducing the electron concentration and conductivity of ZnO [17,18]. When the ZnO film is irradiated with a 365 nm UV lamp, photogenerated electron–hole pairs are produced. As shown in Figure 3a, the optical absorption spectra of ZnO before and after UV irradiation remained the same since the energy required for the band edge absorption of ZnO is slightly higher than the energy of the UV light. Thus, a few photogenerated carriers may be produced. Subsequently, the photogenerated carriers are separated, and the holes migrate to the ZnO surface where the ionized oxygen is trapped and recombined with O^2−^. After recombination, the adsorbed oxygen ions are oxidized to oxygen and could thus desorb from the surface of ZnO, accompanying the release of captured electrons [19]. Furthermore, ZnO NPs have many surface defects in the band gap [20,21]. As light with energy below the band gap illuminates the surface, the electrons become photoexcited to the conduction band, causing electron density to be enhanced, which is associated with negatively charged oxygen ions and oxygen vacancy [17,22]. Therefore, the conductivity of ZnO was effectively improved by UV irradiation. It has been reported that the visible PL emission in ZnO NPs originates from adsorbed O_2_ [23]. Our ZnO NPs film exhibited weak green PL at an excitation wavelength of 280 nm. This green emission became lower after UV irradiation, as shown in Figure 3b. This result indicates the partially adsorbed O_2_ decreased after UV irradiation, which is consistent with the other characteristics above.

The adsorbed oxygen can also create a potential barrier of upward band bending by a depletion region along the ZnO surface, causing a high work function (WF) [24,25,26]. After UV irradiation, the adsorbed oxygen in the ZnO is released, which leads to less band bending and lower WF [27]. As shown in Figure 3c, the WF of the ZnO film exhibited a gradual drop with the exposure of ZnO to a 365 nm UV lamp for 15 min. The WF was kept almost constant with ZnO despite further illumination with UV light, suggesting saturation of the WF shift. The WF of ZnO in the pristine device and the device subjected to UV light were calibrated as 4.27 eV and 3.95 eV, respectively. The barrier height on the surface band bending was 0.32 eV. The reduction in band bending contributed to improvements in the electron transport between adjacent ZnO NPs and the electron injection from ITO into ZnO.

As mentioned above, UV irradiation not only enhanced the conductivity of ZnO, but also increased electron injection. Therefore, with UV irradiation, the device exhibited a significantly larger current and lower turn-on voltage. After UV irradiation treatment, the electron current was considerably enhanced—reaching even two orders of magnitude higher than the hole current (Figure 2a,b). This may help electron-assisted hole injections to improve the charge balance in the QDs layer. It was recently reported that the hole injection is assisted by the enhanced confinement of Coulomb interactions originating from the first electron injection into the QDs [28]. The EQE of QLEDs reached 20.3% with a good charge balance, although the current density of the HOD was approximately three orders of magnitude smaller than that of the EOD. As a result, the UV irradiation enhanced the electron injection to promote the charge balance in the QDs, which led to the improvement of EQE and PE in the QLEDs.

**Figure 4:**
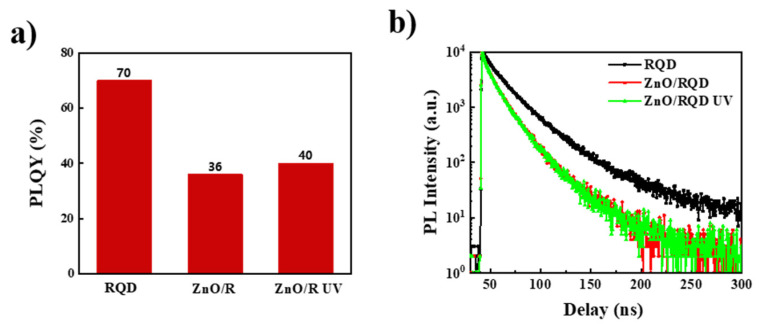
PLQY (**a**) and TRPL lifetime (**b**) of samples quartz/QDs, quartz/ZnO/QDs, and quartz/ZnO/QDs with UV irradiation.

Furthermore, it is also worth understanding whether the UV irradiation affects exciton quenching at the interface between ZnO and QDs. The photoluminescence quantum yield (PLQY) of QDs interfaced with ZnO and the PL lifetime were revealed by time-resolved PL decay curves (Figure 4). The pristine QDs film exhibited a PLQY of 70% and an average TRPL lifetime of 25.8 ns on a quartz substrate. However, the QDs deposited onto the ZnO nanocrystal film showed a lower PLQY of 36% and a shorter average TRPL lifetime of 14.1 ns, which could result from significant interfacial exciton quenching by surface defects that acted as nonradiative recombination centers [29]. The PLQY and TRPL lifetime kept almost the same values after the ZnO/QDs film was irradiated with UV light for 15 min. These results suggest that the UV irradiation treatment did not affect interfacial exciton quenching.

### 3.2. The Effect of Storage Period on Inverted QLEDs

**Figure 5:**
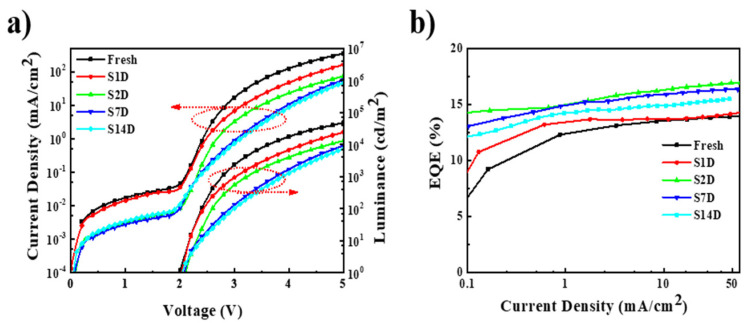
(**a**) The current density–voltage–luminance (J–V–L) characteristics of the QLEDs after storage from 0 to 14 days; (**b**) external quantum efficiency–current density (EQE–J) characteristics of the QLEDs after storage from 0 to 14 days.

Storage aging often occurs in regular devices, and is usually related to the reactions between ZnO and the acid encapsulating adhesive or Al electrode. As the impact of the above two factors on the bottom layer of ZnO should be very small, better storage stability in the inverted device was expected. Here, we analyzed the performance of the devices (encapsulated with UV irradiation for 5 min) after storage for different periods. It can be clearly seen from Figure 5a that the current density of QLEDs decreased significantly after storage, especially after two days of storage. For example, at the driving voltage of 5 V, the current density of the device (S2D) after storage for two days reached 74 mA/cm^2^, which is only about one-fifth that of the pristine device (S0D). This means that the driving voltage at the same brightness will increase significantly after storage. For a brightness of 10,000 cd/m^2^, the driving voltage of the pristine device (S0D) was 4.3 V, but it increased to 4.8 V after two days of storage (S2D), and to 6.1 V after 14 days (S14D). The significant increase of the driving voltage may reduce the stability of the device and affect the display driving circuit. However, it can be observed that the maximum EQE of the device increases after storage from 0–14 days, in comparison to that of the fresh device. The maximum EQE of the device after two days of storage increased to 16.9%, which is 20.7% higher than that of the control device. These changes in performance of the inverted QLEDs may indicate that the device has low storage stability.

**Figure 6:**
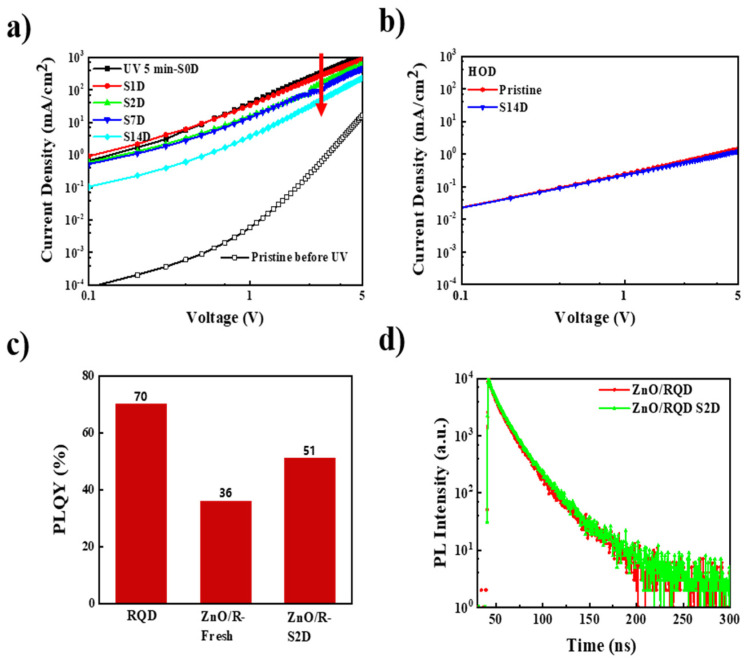
(**a**) J–V curves for the electron-only devices (ITO/ZnO/QDs/TPBi/LiF/Al) before and after storage from 0 to 14 days; (**b**) J–V curves for the hole-only devices before and after storage for 14 days; (**c**) the PLQY and (**d**) TRPL lifetime for the quartz/ZnO/QDs sample before and after storage for 2 days.

Considering that UV irradiation during the device encapsulation process increased the electron current by two orders of magnitude, the decrease in the current of the storage device could originate from the change of ZnO. The EODs and the HODs were fabricated to explore the changes of the device current during storage. As shown in Figure 6a,b, the electron current density decreased with increasing storage time, while the hole current did not show any detectable change. Therefore, during the storage process, the oxygen desorbed on the ZnO surface after UV irradiation could be adsorbed again and the excited electrons from the defect states could decay. As a result, the conductivity of ZnO reduced and the electron injection barrier increased, thereby reducing the electron injection of QLEDs, which in turn led to a reduction in the current density. Combined with the effect of UV light, which improved electron injection, the carrier of QLEDs was more balanced and a higher EQE was obtained. Therefore, during storage, the carrier balance of QLEDs will be destroyed and the EQE will be reduced, as a result of the reduction in electron injection during the storage process. In Figure 5b, it can be seen that the EQE of the device after storage increased compared to the control device. Therefore, there could be other factors improving the performance of QLEDs during storage.

To examine exciton quenching at the interface of ZnO and QD, PLQY and TRPL measurements were carried out on the pristine and stored samples (quartz/ZnO/QDs). After storage for 2 days, the QDs film exhibited a PLQY of 51% and an average TRPL lifetime of 16.0 ns, which are both slightly increased compared to those of the pristine sample (Figure 6c,d). These results reveal that the exciton quenching at the ZnO/QD interface was reduced after storage. It was previously reported that the defects on the ZnO surface were passivated by trace amounts of water, which could be adsorbed on the surface of ZnO NPs or quantum dots in the preparation process [30,31]. The effects of water molecules on the ZnO and QDs still need further detailed characterization.

## 4. Conclusions

We investigated the effects of UV irradiation during the encapsulation process on the efficiency and storage stability of ZnO-based inverted QLEDs. The pristine device exhibited inferior device performance with a high turn-on voltage, low current, and low EQE. The low performance could be attributed to a reduction in ZnO conductivity due to the trace amount of O_2_ in the glovebox being adsorbed on the surface of ZnO, and in turn capturing the free electrons of ZnO. After UV irradiation, the conductivity of ZnO increased and the electron injection barrier decreased. As a result, the inverted QLEDs exhibited higher current, EQE, and PE, possibly due to a more effective charge injection. The EQE and PE of the device after UV irradiation for 15 min was enhanced by 26% and 143%, respectively, compared to the pristine device. Although the UV irradiation demonstrated an advantage in boosting EQE and PE, it could affect the storage stability of the inverted QLEDs. During the storage period, the electron current from ZnO gradually decreased due to re-adsorbed O_2_ and the decay of excited electrons, causing a reduction in device current. However, the inverted QLEDs showed an improvement in the maximum EQE by 20.7% after two days of storage. Our research indicates that the suppression of exciton quenching at the interface of ZnO and QDs during storage could improve the EQE. The effects of UV irradiation and storage stability in inverted QLEDs were demonstrated in this work, offering insights into the fabrication of inverted QLEDs with improved stability.

## Figures and Tables

**Table 1:** The characteristics of the QLEDs with different UV irradiation times.

UV Time	V_on_ (V)	EQE_max_ (%)	PE_max_ (lm/W)	@5 V L (cd/m^2^) J (mA/cm^2^)		@1000 cd/m^2^ V (V) EQE (%)	
Pristine	3.0	12.7	7.9	383	3.2	5.5	12.3
5 min	2.0	14.1	17.0	36,330	263.1	2.8	13.8
10 min	2.0	15.1	17.4	51,175	343.2	2.7	14.5
15 min	2.0	16.0	19.2	65,445	426.9	2.7	15.7
20 min	2.0	15.0	16.8	69,694	474.8	2.7	14.1

## Data Availability

Not applicable.

## References

[1] Alivisatos A.P., Schlamp M.C., Colvin V.L. (1994). Light-emitting Diodes Made from Cadmium Selenide Nanocrystals and a Semiconducting Polymer. Nature.

[2] Dai X., Zhang Z., Jin Y., Niu Y., Cao H., Liang X., Chen L., Wang J., Peng X. (2014). Solution-processed, high-performance light-emitting diodes based on quantum dots. Nature.

[3] Shu Y., Lin X., Qin H., Hu Z., Jin Y., Peng X. (2020). Quantum Dots for Display Applications. Angew. Chem. Int. Ed. Engl..

[4] Cao W., Xiang C., Yang Y., Chen Q., Chen L., Yan X., Qian L. (2018). Highly Stable QLEDs with Improved Hole Injection via Quantum Dot Structure Tailoring. Nat. Commun..

[5] Shen H., Gao Q., Zhang Y., Lin Y., Lin Q., Li Z., Chen L., Zeng Z., Li X., Jia Y. (2019). Visible Quantum Dot Light-emitting Diodes with Simultaneous High Brightness and Efficiency. Nat. Photonics.

[6] Li X., Zhao Y.B., Fan F., Levina L., Liu M., Quintero-Bermudez R., Gong X., Quan L.N., Fan J., Yang Z. (2018). Bright Colloidal Quantum Dot Light-emitting Diodes Enabled by Efficient Chlorination. Nat. Photonics.

[7] Pu C., Dai X., Shu Y., Zhu M., Deng Y., Jin Y., Peng X. (2020). Electrochemically-stable Ligands Bridge the Photoluminescence-electroluminescence Gap of Quantum Dots. Nat. Commun..

[8] Zhang Z., Ye Y., Pu C., Deng Y., Dai X., Chen X., Chen D., Zheng X., Gao Y., Fang W. (2018). High-Performance, Solution-Processed, and Insulating-Layer-Free Light-Emitting Diodes Based on Colloidal Quantum Dots. Adv. Mater..

[9] Rhee S., Chang J.H., Hahm D., Jeong B.G., Kim J., Lee H., Lim J., Hwang E., Kwak J., Bae W.K. (2020). Tailoring the Electronic Landscape of Quantum Dot Light-Emitting Diodes for High Brightness and Stable Operation. ACS Nano.

[10] Song J.J., Wang O., Shen H.B., Lin Q.L., Li Z.H., Wang L., Zhang X.T., Li L.S. (2019). Over 30% External Quantum Efficiency Light-Emitting Diodes by Engineering Quantum Dot-Assisted Energy Level Match for Hole Transport Layer. Adv. Funct. Mater..

[11] Li X., Lin Q., Song J., Shen H., Zhang H., Li L.S., Li X., Du Z. (2019). Quantum-Dot Light-Emitting Diodes for Outdoor Displays with High Stability at High Brightness. Adv. Opt. Mater..

[12] Wang L., Lin J., Hu Y., Guo X., Lv Y., Tang Z., Zhao J., Fan Y., Zhang N., Wang Y. (2017). Blue Quantum Dot Light-Emitting Diodes with High Electroluminescent Efficiency. ACS Appl. Mater. Interfaces.

[13] Xiang C., Wu L., Lu Z., Li M., Wen Y., Yang Y., Liu W., Zhang T., Cao W., Tsang S.W. (2020). High Efficiency and Stability of Ink-Jet Printed Quantum Dot Light Emitting Diodes. Nat. Commun..

[14] Acharya K.P., Titov A., Hyvonen J., Wang C., Tokarz J., Holloway P.H. (2017). High Efficiency Quantum Dot Light Emitting Diodes from Positive Aging. Nanoscale.

[15] Su Q., Sun Y., Zhang H., Chen S. (2018). Origin of Positive Aging in Quantum-Dot Light-Emitting Diodes. Adv. Sci..

[16] Chen D., Chen D., Dai X., Zhang Z., Lin J., Deng Y., Hao Y., Zhang C., Zhu H., Gao F. (2020). Shelf-Stable Quantum-Dot Light-Emitting Diodes with High Operational Performance. Adv. Mater..

[17] Li Q.H., Gao T., Wang Y.G., Wang T.H. (2005). Adsorption and Desorption of Oxygen Probed from ZnO Nanowire Films by Photocurrent Measurements. Appl. Phys. Lett..

[18] Bao J., Shalish I., Su Z., Gurwitz R., Capasso F., Wang X., Ren Z. (2011). Photoinduced Oxygen Release and Persistent Photoconductivity in ZnO Nanowires. Nanoscale Res. Lett..

[19] Tudose I.V., Horvath P., Suchea M., Christoulakis S., Kitsopoulos T., Kiriakidis G. (2007). Correlation of ZnO Thin Film Surface Properties with Conductivity. Appl. Phys. A-Mater..

[20] Zhang S.B., Wei S.H., Zunger A. (2001). Intrinsic N-type Versus P-type Doping Asymmetry and the Defect Physics of ZnO. Phys. Rev. B.

[21] Djurisic A.B., Leung Y.H. (2006). Optical Properties of ZnO Nanostructures. Small.

[22] Lany S., Zunger A. (2005). Anion Vacancies as a Source of Persistent Photoconductivity in II-VI and Chalcopyrite Semiconductors. Phys. Rev. B.

[23] Ma Y., Choi T.W., Cheung S.H., Cheng Y., Xu X., Xie Y.M., Li H.W., Li M., Luo H., Zhang W. (2019). Charge Transfer-induced Photoluminescence in ZnO Nanoparticles. Nanoscale.

[24] Li Y.B., Della Valle F., Simonnet M., Yamada I., Delaunay J.J. (2009). Competitive Surface Effects of Oxygen and Water on UV Photoresponse of ZnO Nanowires. Appl. Phys. Lett..

[25] Bao Q.Y., Liu X.J., Xia Y.X., Gao F., Kauffmann L.D., Margeat O., Ackermann J., Fahlman M. (2014). Effects of Ultraviolet Soaking on Surface Electronic Structures of Solution Processed ZnO Nanoparticle Films in Polymer Solar Cells. J. Mater. Chem. A.

[26] Raoufi M., Hormann U., Ligorio G., Hildebrandt J., Patzel M., Schultz T., Perdigon L., Koch N., List-Kratochvil E., Hecht S. (2020). Simultaneous Effect of Ultraviolet Radiation and Surface Modification on the Work Function and Hole Injection Properties of ZnO Thin Films. Phys. Status Solidi A.

[27] Li G.D., Meng L.X., Zhu X.F., Gao W.H., Qin Y., Chen L.W. (2018). Clarifying the High On/Off Ratio Mechanism of Nanowire UV Photodetector by Characterizing Surface Barrier Height. Nanoscale.

[28] Deng Y., Lin X., Fang W., Di D., Wang L., Friend R.H., Peng X., Jin Y. (2020). Deciphering Exciton-generation Processes in Quantum-dot Electroluminescence. Nat. Commun..

[29] Sun Y., Jiang Y., Peng H., Wei J., Zhang S., Chen S. (2017). Efficient Quantum Dot Light-emitting Diodes with a Zn_0.85_Mg_0.15_O Interfacial Modification Layer. Nanoscale.

[30] Zhang W., Chen X., Ma Y., Xu Z., Wu L., Yang Y., Tsang S.W., Chen S. (2020). Positive Aging Effect of ZnO Nanoparticles Induced by Surface Stabilization. J. Phys. Chem. Lett..

[31] Tavasoli E., Guo Y.J., Kunal P., Grajeda J., Gerber A., Vela J. (2012). Surface Doping Quantum Dots with Chemically Active Native Ligands: Controlling Valence without Ligand Exchange. Chem. Mater..

